# Care model selection for older adult stroke survivors with disabilities: insights from the eighth wave of CLHLS data and influencing factors

**DOI:** 10.3389/fpubh.2024.1404537

**Published:** 2024-06-11

**Authors:** Liping Xiang, Qin Liu, Zijuan Shi, Li Zhang, Li Wu, Yuqin Chen

**Affiliations:** ^1^North Sichuan Medical College, Nanchong, China; ^2^Affiliated Hospital of North Sichuan Medical College, Nanchong, China

**Keywords:** stroke, disabled older adult, care model, CLHLS, the influencing factors

## Abstract

**Background:**

Analyzing the differences in caregiving models for disabled older adult individuals after stroke and the influencing factors, to provide a basis for addressing relevant social demographic issues.

**Methods:**

The older adult diagnosed with stroke were screened from the Chinese Geriatric Health Survey (CLHLS), and were further divided into subgroups of disability, which was based on their ability of or whether they need help in performing activities such as dressing, bathing, eating, toileting or bowel and bladder control using the international common Katz scale. The care model was divided into formal care, informal care and home care. Multivariate logistic regression was used to screen the influencing factors of the choice of care model for the disabled older adult after stroke.

**Results:**

The results of univariate analysis showed that there were statistical differences in the choice of care mode among different ages, household registration types, number of children, years of education, degree of disability, community services, retirement pension, marital status and medical insurance. Multiple logistic regression showed that, The rural older adult with more children, shorter education years, living with spouse and no help from community tend to choose informal care. Older adult people with higher levels of education, urban household registration, and access to community services are more likely to choose formal care. Older adult women with multiple children are more likely to receive care from their children.

**Conclusion:**

In the future, vigorous support for the development of formal caregiving institutions and the improvement of the management system of formal caregiving will help enhance the subjective initiative of disabled older adult individuals in choosing caregiving models and alleviate the burden of family caregiving.

## 1 Introduction

The 2019 Global Disease Burden (GDB) showed that stroke remains the second leading cause of death globally and the third leading cause of death and disability. Moreover, China has the highest lifetime incidence of stroke, the incidence of stroke has been reported to be 345.1 per 100,000 individuals (1), and there were consistent annual increases in the prevalence of stroke in China between 2013 and 2019, from 2.28 to 2.58% (2). Stroke is associated with high morbidity, a high prevalence of disability, a high mortality rate, a high rate of recurrence, and a significant economic burden (3). Most stroke survivors experience forms of disability, such as dysphagia, aphasia, social impairment, and cognitive abnormalities, which tend to persist (4). Owing to cultural factors, such as “filial piety” values, and the limited medical resources available for rehabilitation in China, most patients who experience stroke are discharged from hospital and family members assume the responsibility for care (5). Older survivors of stroke are at significantly higher risks of events including falls, aspiration pneumonia, getting lost or wandering, and pressure ulcers than other older individuals (6). Unlike for other progressive diseases, someone who has experienced a stroke has unpredictable care needs (7), and therefore caregivers must be adequately prepared for the discharge of the patient from hospital and capable of providing long-term care (8, 9), which often involves dedicating themselves entirely to this role. Family members inevitably experience the impact of the disease themselves (10), and the prolonged intensive caregiving duties can negatively affect their mental health and quality of life, in addition to imposing a significant financial burden (11, 12). In particular, partners and spouses are at a high risk of anxiety and depression (13). Thus, stroke not only has significant effects on the patient’s quality of life (14), but also places substantial burdens on both families and society as a whole (15).

Because China has the largest population of people with disabilities in the world, it has a larger demand for care from older people with disabilities than any other country. Therefore, pension and care issues have always been important nationally (16). Kinship serves as the foundation of caregiving (17), but with the aging of the first generation of only children’s parents in China, children are gradually becoming the primary potential caregivers for the older adult population (18). Moreover, Chinese culture has long emphasized the importance of raising children to support their parents in their old age. In China, the establishment of formal nursing institutions is relatively scattered and the development is not yet mature enough. Furthermore, not all older adult individuals can afford the associated fees (19, 20), family size and economic status still largely determine the approach to caregiving in older people with disabilities (21, 22).

The caregiving model can be categorized as formal care or informal care, according to whether or not professionals are involved. Formal care involves the provision of paid professional assistance by institutions and nursing professionals, while informal care comprises unpaid support offered by family members, friends, or social workers (23). In China, 83.0% of older people with disabilities choose to have home-based care (24), but the care of older people with stroke-related disability is not solely a family matter, but also a societal concern (16). As families continue to shrink in size, the following hierarchy is generally followed with respect to the provision of care for older individuals with disabilities in China: care by a spouse, followed by care by a child, and finally care by other relatives or paid caregivers/institutions if the first two cannot meet the requirements of the patient adequately (25).

In the present study, we have analyzed the existing caregiving models and the factors influencing their selection for the care of older individuals with stroke-related disability, based on hierarchical compensation theory. For this purpose, older people residing in nursing homes were categorized as receiving formal care, while those receiving care from family members, relatives, friends, or neighbors were categorized as receiving informal care. In addition, older individuals who were receiving care from professional visitors and social services were classified as receiving home care. We compared people receiving formal, informal, or home-based with respect to their demographics and the factors influencing the choice of these types of care.

## 2 Materials and methods

### 2.1 Data sources

We analyzed data from the Chinese Longitudinal Healthy Longevity Survey (CLHLS), organized by the Center for Healthy Aging and Development of Peking University/National Institute of Development Research. The survey covered 23 provinces, municipalities, and autonomous regions in China, and the participants were people aged ≥65 years and their children aged 35–64 years. A total of 15,874 people aged ≥65 years were interviewed during the eighth follow-up survey in 2017–2018. The investigators were registered with and given access to the data platform.

### 2.2 Selection of patients and classification of disability

The key English word “stroke” was used to screen older adult patients with stroke. The types of stroke were unspecific in the CLHLS database while 70% of strokes were acute ischemic stroke in China (26). This study analyzed data exclusively from disabled older adult individuals diagnosed with stroke. The Activity of Daily Living (ADL) scale was used to quantify the need for help with six daily activities (dressing, bathing, eating, going to the toilet, bowel and bladder control, and indoor activities). If help was required with one or more of these items, the presence of disability was recorded, and the degree of disability was classified according to the number of items for which help was required. If help was required for 1–2 items, 3–4 items, or 5–6 items, the participants were classified as having mild, moderate, or severe disability, respectively (27).

### 2.3 Objectives and methods

This study analyzed disabled older adult individuals with stroke, excluding those who were fully capable of independently performing daily activities, and removed samples with missing key variables. Ultimately, data from 629 disabled older adult stroke patients were included for analysis. Microsoft Excel was used to compile and organize the data, and SPSS v.26.0 (IBM, Inc., Armonk, NY, United States) was used for data analysis. Missing data were processed using two methods: data with missing values in variables exceeding 20% were deleted, and simple imputation was applied to address other missing data. Mode imputation was used for categorical data, and median or mean imputation was used for continuous data. Mean ± standard deviation and median (interquartile range) are used to summarize continuous data, and variance analysis or a non-parametric test was used to compare datasets. Percentages or ratios are used to describe categorical data, and the χ^2^ test was used to compare the datasets. Multivariate logistic regression was used to identify factors influencing the choice of care model. The tests were all two-sided, and *p* < 0.05 was considered to represent statistical significance.

## 3 Results

### 3.1 General condition of the participants

A total of 629 older people with stroke-related disability were included, of whom 478 (80.0%) were receiving informal care, 95 (15.1%) were receiving formal care, and 56 (8.9%) were receiving home care. They comprised 269 men (42.8%) and 360 women (57.2%); 316 (50.2%) were urban residents and 333 (49.8%) were rural residents. Their mean age was 90.5 ± 9.8 years, and their mean duration of education was 3.99 ± 7.99 years. There were 213 participants (33.9%) with mild disability, 130 (20.7%) with moderate disability, and 286 (45.5%) with severe disability. Furthermore, 292 participants (46.4%) received community services and 337 (53.6%) did not. The mean numbers of children and sons were 3.63 ± 1.83 and 1.79 ± 1.25, respectively. In addition, 173 (27.5%) were married and living with their spouse, and 456 (72.5%) were widowed, divorced, or married but not living with their spouse. Finally, 254 participants (40.4%) had a retirement pension; 161 (25.6%) had a pension funds, 506 (80.5%) had medical insurance (including free medical insurance, urban employee/resident medical insurance), new rural cooperative medical insurance, or commercial medical insurance, and 163 (25.9%) had pension insurance.

### 3.2 Results of the univariate analysis of the various care models

The univariate analysis showed that there were significant differences in the participation in the various care models with respect to age, household registration type, number of living children, the duration of education, the degree of disability, community service status, pension status, marital status, and medical insurance (*p* < 0.05), as shown in Table 1.

**Table 1:** A comparison of basic conditions of care models for disabled older adult stroke patients.

Variables	Formal care	Informal care	Home care	Statistical value	*P*
Sex [*n* (%)]				0.36	0.835
Male	42 (44.21)	205 (42.89)	22 (39.29)		
Female	53 (55.79)	273 (57.11)	34 (60.71)		
Age ( $\bar{x}\pm s$ )	89.26 ± 9.82	90.35 ± 9.73	93.29 ± 9.45	6.91	0.032^*^
Residence [*n* (%)]				107.44	<0.001^**^
City/town	86 (90.53)	185 (38.70)	45 (80.36)		
Rural	9 (9.47)	293 (61.30)	11 (19.64)		
Number of children ( $\bar{x}\pm s$ )	2.64 ± 1.76	3.83 ± 1.76	3.54 ± 1.74	35.86	<0.001^**^
Education (years) ( $\bar{x}\pm s$ )	6.44 ± 12.92	3.01 ± 5.40	8.17 ± 12.41	35.36	<0.001^**^
Disability [*n* (%)]				31.61	<0.001^**^
Mild disability	17 (17.89)	183 (38.28)	13 (23.21)		
Moderate disability	12 (12.63)	105 (21.97)	13 (23.21)		
Severe disability	66 (69.47)	190 (39.75)	30 (53.57)		
Community services [*n* (%)]				14.85	0.001^*^
No	34 (35.79)	274 (57.32)	29 (51.79)		
Yes	61 (64.21)	204 (42.68)	27 (48.21)		
Retirement pension [*n* (%)]				61.48	<0.001^**^
No	33 (34.74)	326 (68.20)	16 (28.57)		
Yes	62 (65.26)	152 (31.80)	40 (71.43)		
Pension funds [*n* (%)]				3.16	0.164
No	75 (78.95)	347 (72.59)	46 (82.14)		
Yes	20 (21.05)	131 (27.41)	10 (17.86)		
Pension insurance [*n* (%)]				3.97	0.137
No	75 (78.95)	345 (72.18)	46 (82.14)		
Yes	20 (21.05)	133 (27.82)	10 (17.86)		
Marital status [*n* (%)]				26.37	<0.001^**^
Married and living with their spouse	10 (10.53)	156 (32.64)	7 (12.50)		
Other marital status	85 (89.47)	322 (67.36)	49 (87.50)		
Medical insurance [*n* (%)]				11.92	0.003^*^
No	29 (30.53)	79 (16.53)	15 (26.79)		
Yes	66 (69.47)	399 (83.47)	41 (73.21)		
Living standard (self-evaluated) ( $\bar{x}\pm s$ )	3.72 ± 0.72	3.83 ± 0.75	3.93 ± 0.81	3.48	0.175
Health status (self-evaluated) ( $\bar{x}\pm s$ )	2.98 ± 0.64	2.86 ± 0.86	2.93 ± 0.76	2.30	0.316

^*^P < 0.05; ^**^p < 0.01; P, *p*-value; community services: various forms of support and assistance provided in the residential communities, including healthcare, daily living aid and social activities; retirement pension/fund: income after retirement; pension insurance: expenditure for income after retirement. Other marital situations include widowhood, divorce, and being married but not cohabiting; self-rated quality of life and self-rated health scores are converted from very poor, poor, fair, good, very good according to the Likert 5-point rating scale.

### 3.3 Results of the multiple logistic regression analysis of the various care models

We used the care mode as the dependent variable and the informal care model as the control group, and the factors identified as significant on univariate analysis were included in the multiple logistic regression model. The result of a parallel test was *p* < 0.001, suggesting that the disordered multiple logistic regression analysis could be used. However, the generalized variation-inflation factors for age and the degree of disability were 22.3 and 329.1, respectively, suggesting that the model may involve collinearity. Therefore, the unordered multi-class logistic regression analysis was performed again after removing these two variables. Compared with the informal care model, having more children and more female children, having medical insurance, and living with a spouse were found to be protective factors for the formal care model (*p* < 0.05); whereas higher education level, urban household registration, and having community help were found to be risk factors (*p* < 0.05). This implies that rural-dwelling older people with more children, who had a shorter duration of education, who lived with their spouse, and who had no help from the community tended to choose the informal care model. Compared with the informal care model, a longer duration of education, an urban household registration, other marital status (including widowed, divorced, or married but not living with their spouse), and having a pension were risk factors for the home care model (*p* < 0.05). This implies that urban-dwelling older people with a higher educational level, who were not living with their spouse, and who had a retirement pension were more likely to choose the home care model. See Table 2 for further details.

**Table 2:** Analysis of factors influencing care models for disabled older adult stroke patients.

Variables	β	SE	Waldχ^2^	*P*	OR (95%CI)
Formal care					
Number of children	−0.35	0.08	−4.29	<0.001^**^	0.70 (0.60, 0.82)
Education	0.03	0.02	1.99	0.046^*^	1.03 (1.00, 1.07)
Residence (Compared with Rural Areas)					
City/Town	2.29	0.41	5.54	<0.001^**^	9.92 (4.40, 22.37)
Marital status (Compared with other marital status)					
Married and living with their spouse	−1.72	0.38	−4.54	<0.001^**^	0.18 (0.09, 0.38)
Medical insurance (Compared with no medical insurance)					
Yes	−0.61	0.31	−2.00	0.045^*^	0.54 (0.30, 0.99)
Retirement pension (Compared with no retirement pension)					
Yes	0.24	0.31	0.78	0.433	1.27 (0.70, 2.32)
Community services (Compared with no community services)					
Yes	0.89	0.27	3.32	0.001^*^	2.44 (1.44, 4.12)
Home care					
Number of children	−0.04	0.09	−0.45	0.065	0.96 (0.80, 1.15)
Education	0.05	0.02	3.06	0.002^*^	1.05 (1.02, 1.08)
Residence (Compared with Rural Areas)					
City/Town	1.11	0.43	2.60	<0.001^**^	3.05 (1.31, 7.07)
Marital status (Compared with other marital status)					
Married and living with their spouse	−1.52	0.45	−3.43	<0.001^**^	0.22 (0.09, 0.52)
Medical insurance (Compared with no medical insurance)					
Yes	−0.56	0.35	−1.59	0.112	0.57 (0.29, 1.14)
Retirement pension (Compared with no retirement pension)					
Yes	0.99	0.39	2.57	0.010^*^	2.72 (1.27, 5.82)
Community services (Compared with no community services)					
Yes	0.39	0.31	1.29	0.199	1.48 (0.81, 2.71)

^*^P < 0.05; ^**^P < 0.01; Waldχ2, Wald Chi-Square value; P, *p*-value; β, regression coefficient; SE, standard error; OR, odds ratio; CI, confidence interval. Other marital situations include widowhood, divorce, and being married but not cohabiting; community services: various forms of support and assistance provided in the residential communities, including healthcare, daily living aid and social activities; retirement pension: income after retirement.

### 3.4 Subgroup analysis of the use of the informal care model

The participants who received informal care were next further studied. The caregivers for these participants were categorized according to their caregiver (108 received care from a spouse, 366 received care from children, and 4 received care from people with other relationships), and then their situations were compared. Because there were only four participants who received care from people other than their spouse or children, this group was not included in the comparison. We found that the informal care model used was related to sex, age, the number of existing children, the duration of education, living standard (self-evaluated), health status (self-evaluated). See Table 3 for details.

**Table 3:** Comparison of basic information on the informal care models for disabled older adult stroke patients.

Variables	Care from children	Care from spouse	Statistical value	*P*
Sex [*n* (%)]			61.71	<0.001^**^
Male	120 (32.79)	82 (75.93)		
Female	246 (67.21)	26 (24.07)		
Age ( $\bar{x}\pm s$ )	92.97 (8.43)	81.16 (8.17)	33,134	<0.001^**^
Residence [*n* (%)]			0.00	0.994
City/town	140 (38.25)	42 (38.89)		
Rural	226 (61.75)	66 (61.11)		
Marital status [*n* (%)]			265.78	<0.001^**^
Married and living with their spouse	50 (13.66)	106 (98.15)		
Other marital status	316 (86.34)	2 (1.85)		
Number of children [M(Q1, Q3)]	4.04 (1.77)	3.23 (1.53)	18.43	<0.001^**^
Education (years) ( $\bar{x}\pm s$ )	2.59 (5.62)	4.31 (4.26)	23.35	0.004^*^
Disability [*n* (%)]			5.49	0.064
Mild disability	130 (35.52)	51 (47.22)		
Moderate disability	81 (22.13)	23 (21.30)		
Severe disability	155 (42.35)	34 (31.48)		
Community services [*n* (%)]			0.01	0.916
No	211 (57.65)	61 (56.48)		
Yes	155 (42.35)	47 (43.52)		
Pension funds [*n* (%)]			0.21	0.645
No	268 (73.22)	76 (70.37)		
Yes	98 (26.78)	32 (29.63)		
Retirement pension [*n* (%)]			3.84	0.050
No	259 (70.77)	65 (60.19)		
Yes	107 (29.23)	43 (39.81)		
Pension insurance [*n* (%)]			0.12	0.728
No	266 (72.68)	76 (70.37)		
Yes	100 (27.32)	32 (29.63)		
Medical insurance [*n* (%)]			0.00	0.999
No	60 (16.39)	18 (16.67)		
Yes	306 (83.61)	90 (83.33)		
Living standard (self-evaluated) ( $\bar{x}\pm s$ )	3.87 (0.74)	3.64 (0.75)	11.33	<0.001^**^
Health status (self-evaluated) ( $\bar{x}\pm s$ )	2.93 (0.86)	2.59 (0.77)	16.91	<0.001^**^

^*^P < 0.05; ^**^P < 0.01; P, *p*-value; community services: various forms of support and assistance provided in the residential communities, including healthcare, daily living aid and social activities; retirement pension/fund: income after retirement; pension insurance: expenditure for income after retirement. Other marital situations include widowhood, divorce, and being married but not cohabiting; self-rated quality of life and self-rated health scores are converted from very poor, poor, fair, good, very good according to the Likert 5-point rating scale.

To further explore the factors associated with the type of informal care, a binary logistic regression equation was fitted, using care by children as the control group. We found that older women with more children were more likely to be cared for by their children. See Table 4 for further details.

**Table 4:** Analysis of factors influencing informal care mode for disabled older adult stroke patients.

Variables	β	SE	Waldχ^2^	*P*	OR (95%CI)
Female	−2.16	0.33	−6.61	<0.001^**^	0.11 (0.06,0.21)
Age	−0.16	0.02	−8.36	<0.001^**^	0.85 (0.06,0.88)
Number of children	−0.28	0.09	−3.03	0.002^*^	0.76 (0.63,0.90)
Education	−0.01	0.03	−0.24	0.814	0.99 (0.92,1.04)
Living standard (self-evaluated)	−0.14	0.21	−0.70	0.487	0.87 (0.58,1.30)
Health status (self-evaluated)	−0.11	0.19	−0.57	0.568	8.98 (0.62,1.30)

^*^P < 0.05; ^**^P < 0.01; Waldχ^2^, Wald Chi-Square value; P, *p*-value; β, regression coefficient; SE, standard error; OR, odds ratio; CI, confidence interval.

## 4 Discussion

### 4.1 Older people with many children who live with their spouse and have medical insurance are more likely to receive informal care

Informal care is unpaid, and is therefore rooted in love and a feeling of responsibility (28). The Chinese culture has a fundamental belief in raising children to provide care during old age, and therefore families with ample human and social resources prefer informal care. Furthermore, older individuals with multiple children have greater access to informal care resources (29). Therefore, informal care is frequently considered to be a substitute for formal care (28). However, as China’s economy and society continue to develop, families are becoming smaller and more nuclear. Younger generations may struggle to balance their work and familial obligations, resulting in a more limited ability to provide adequate care for their older adults. As a result, spouses are becoming the primary source of care, and their capability ultimately determine whether patients who experience stroke can be discharged from hospital (30). This is consistent with the findings of Nguyen (31). Medical insurance plays an important role in determining whether older individuals receive informal care, probably because it covers hospitalization expenses, which reduces the financial burden on families, enabling them to be able to afford home care costs (32). The study by Gao (33) showed that older people with disabilities who live in rural areas tend to adopt a “rotation” method when they have multiple children, allowing their children either to visit or take them to their own homes for personal care. This approach reduces the burden on individual children while permitting personalized attention from loved ones. Therefore, those who have many children are more willing and better equipped to receive informal care.

### 4.2 Urban-dwelling older people with a higher educational level, a higher level of disability, and access to community services are more likely to receive formal care

In the 1980s, as a result of the “family planning” policy (34), couples were restricted to having only one child. The educational level of older adults determines their income source and socioeconomic status, with most being employed in formal work and therefore being constrained by the policy, such that they predominantly have a single child. Such single children are unable to share responsibility for older adult care, unlike individuals from multi-child families, and therefore can only provide limited care and economic assistance (35). Older individuals with higher levels of education are more likely to receive formal care, to be able to access information and resources related to such care, and to have the financial means to support it.

The community services provided indirectly reflect the living standards of older adults. In China, middle-and high-grade communities provide corresponding levels of property management for their residents. Therefore, older individuals who have access to these community services have better living conditions and higher economic status. The care institutions for older people in China are primarily concentrated in urban areas (36), meaning that urban-dwelling seniors have better access to formal care than their rural counterparts. However, severe disability necessitates the investment of more time and energy into daily caregiving, and this not only involves physical exertion but also tests the psychological well-being of the caregivers. Caring for older people with severe disabilities is more likely to cause fatigue, anxiety, depression, and other negative emotions that significantly affect the quality of life of caregivers (37, 38). The sequelae following a stroke encompass a spectrum of manifestations, ranging from dysphagia, limb paralysis, and aphasia to the onset of dementia and beyond (39), caregivers need specific professional knowledge and skills in order to provide optimal care for such older people, and those who feel that their partners or relatives are unable to fully manage their lives after a stroke frequently experience feelings of confusion and uncertainty after their partner or relative is discharged from hospital (40). For various reasons, family members may choose to place their older loved ones into professional nursing institutions so that they can receive expert care (41).

### 4.3 Urban-dwelling older people with disabilities who have higher levels of education, receive a pension, and are divorced or widowed are more likely to live at home

Home-based care options include the use of community-based services or assistance provided by caregivers or professional visitors. Older individuals who receive a pension are more financially capable of affording such care. Compared to those who have living spouses, older individuals who do not are less likely to use home-based care, because they are reluctant to disrupt their children’s lives. Previous studies have shown that in the absence of spousal care, some older individuals exhibit a “partition effect” that causes a deviates from the hierarchical compensation theory of caregiving. This means that without spousal support, certain seniors choose professional in-home services or institutional care, instead of relying on their children for assistance (42), which is consistent with the findings of the present study. Furthermore, previous studies have demonstrated a significant substitution effect with professional in-home care services and institutional care among older individuals with disabilities. Specifically, as the economic conditions of the country have improved, there has been no significant change in the likelihood of choosing institutional care, but there has been a notable increase in the use of professional in-home services (43). There has been a shift in caring practices for older people with disabilities from home-based care toward institutional care, and then toward de-institutionalization (44). Whether choosing home-based care—professional visitors and social services provided at home, or formal care—residing in nursing homes, both require the older adult to have sufficient financial means to afford these services and a willingness to accept this care model. This is because both of these care models contradict China’s traditional belief in filial piety and caregiving responsibilities. Therefore, although the conclusion suggests that older adult individuals choosing home-based care and formal care share common characteristics—higher education levels and urban residency, this conclusion is entirely consistent with the current situation in China.

### 4.4 Selection of care by children or a spouse under the informal care model

Men are more likely to receive care from their spouse than women, which may be explained by the tradition in China that women are the more caring, considerate, and gentle sex. In most Chinese families, women are referred to as “housewives,” and are thought to be more suited to the role of caregivers. Thus, older women who have multiple children are more likely to be cared for by their children (21). However, the study by Wu et al. (41) showed that women are more likely to receive social care. These contradictory findings may be explained by differences in the classification method used in these studies; older people receiving institutional care were classified as receiving formal care in the latter study.

Furthermore, older women with stroke-related disabilities need more care than those with other types of disability, because of their lesser ability to take care of themselves. The required care is not only physical, but also includes the need for spiritual comfort. Because many women will have put more effort into raising their children than their partners, it may be that their children will be more inclined to take care of their mothers when they need care. Moreover, many older women will have been widowed. Therefore, children may not choose to install their parent in a nursing home, but may instead choose to take care of them themselves (45).

There is a paucity of data on the selection of different cares with a specific disease. The present study, using a Chinese database updated in 2018, demonstrated that 15.1, 80.0 and 8.9% of older adult patients with strokes chose formal care, informal care and home care, respectively. Using the same database updated in 2014, another study showed that 3.6 and 88.4% of all disabled older adult patients, irrespective of causes, chose formal care and informal care, respectively (46). It was predicted that formal care might be more popular among older adult people in the future (47).

## 5 Conclusion

The choice of care model for older people with stroke-related disabilities is affected by many factors, including personal, family, social, and financial factors. At present, such individuals generally receive informal care from family members in China, and their spouses are the typical principal caregivers. Furthermore, owing to the relatively early stage of development of care institutions in China, bed shortages, high fees, and financial constraints, formal care institutions have not been fully accepted by the public and the system is not of a high standard. However, policies are expected to be introduced that will improve the management of formal care institutions, develop social care, and promote the implementation of long-term care insurance, which should help overcome the financial or human resource limitations to the care of older people with disabilities and their families, and permit respite care to be provided. Owing to its various sequelae, stroke presents great challenges in the care of older people. Communities should play a leading role in organizing volunteers to provide relevant knowledge about, and assistance with, care, and help caregivers provide more professional care to such older people in their homes, to reduce the risk of complications and adverse events and improve their quality of life.

## Data availability statement

The datasets presented in this study can be found in online repositories. The names of the repository/repositories and accession number(s) can be found below: http://opendata.pku.edu.cn. Further inquiries can be directed to the corresponding author.

## Ethics statement

Written informed consent from the patients/participants or patients'/participants' legal guardian/next of kin was not required to participate in this study in accordance with the national legislation and the institutional requirements.

## Author contributions

LX: Writing – original draft, Writing – review & editing, Data curation, Formal analysis, Resources. QL: Writing – original draft, Writing – review & editing. ZS: Methodology, Writing – review & editing. LZ: Validation, Writing – review & editing. LW: Conceptualization, Writing – review & editing. YC: Project administration, Writing – review & editing.
